# Respiratory Tract Oncobiome in Lung Carcinogenesis: Where Are We Now?

**DOI:** 10.3390/cancers15204935

**Published:** 2023-10-11

**Authors:** Karolina H. Czarnecka-Chrebelska, Jacek Kordiak, Ewa Brzeziańska-Lasota, Dorota Pastuszak-Lewandoska

**Affiliations:** 1 Department of Biomedicine and Genetics, Medical University of Lodz, Mazowiecka 5, 92-215 Lodz, Poland; 2 Department of Thoracic, General and Oncological Surgery, Medical University of Lodz, 90-151 Lodz, Poland; 3 Department of Microbiology and Laboratory Medical Immunology, Medical University of Lodz, Pomorska 251, 90-151 Lodz, Poland; dorota.pastuszak-lewandoska@umed.lodz.pl

**Keywords:** lung cancer, microbiota, oncobiome, host–microbiota interactions

## Abstract

**Simple Summary:**

Research has clearly shown a connection between the respiratory tract microbiome and lung cancer. The composition and metabolism of the bacterial community in lung cancer patients differ from those in healthy individuals. Further large-scale studies are needed to understand the microbiome’s role in lung cancer, including identifying bacterial species, deciphering mechanisms and relationships with the macro-organisms, and addressing analysis-related issues. Large-scale research is also needed on the lung mycobiome and virome. Identifying microorganisms involved in oncogenic processes could improve lung cancer patient screening, diagnosis, and therapeutic options. This review presents the current state of knowledge on the role of the respiratory tract microbiome in lung carcinogenesis. We highlight what we know and what we don’ yet know about the human lung oncobiome.

**Abstract:**

The importance of microbiota in developing and treating diseases, including lung cancer (LC), is becoming increasingly recognized. Studies have shown differences in microorganism populations in the upper and lower respiratory tracts of patients with lung cancer compared to healthy individuals, indicating a link between dysbiosis and lung cancer. However, it is not only important to identify “which bacteria are present” but also to understand “how” they affect lung carcinogenesis. The interactions between the host and lung microbiota are complex, and our knowledge of this relationship is limited. This review presents research findings on the bacterial lung microbiota and discusses the mechanisms by which lung-dwelling microorganisms may directly or indirectly contribute to the development of lung cancer. These mechanisms include influences on the host immune system regulation and the local immune microenvironment, the regulation of oncogenic signaling pathways in epithelial cells (causing cell cycle disorders, mutagenesis, and DNA damage), and lastly, the MAMPs-mediated path involving the effects of bacteriocins, TLRs signaling induction, and TNF release. A better understanding of lung microbiota’s role in lung tumor pathology could lead to identifying new diagnostic and therapeutic biomarkers and developing personalized therapeutic management for lung cancer patients.

## 1. Introduction

This is a review paper summarizing the current knowledge on the bacterial community inhabiting the lungs and its role in lung oncogenesis. Based on the latest literature, we describe the role that the lung microbiome may play in initiating and maintaining the cancer process in the lungs. We try to answer the following questions. What is the difference between the lung microbiome in healthy people and in cancer patients? What factors lead to dysbiosis of the normal lung microbiome? Most importantly, by what mechanisms do the microbes themselves influence and disrupt cellular processes? It is important to answer not only the question “who is there”, but also “what they are doing”, i.e., how bacteria influence lung carcinogenesis. In this regard, we pay particular attention to immunological mechanisms, as it is widely recognized that chronic inflammation plays an important role in carcinogenesis, including lung cancer. We also look at what bacterial species can be considered potential biomarkers for lung cancer. Have they already been identified and confirmed? It is so important, as they may have diagnostic and/or prognostic utility. They can also be the target of therapeutic and adjuvant procedures, such as the engineering of the microbiome with antibiotics, antibodies, or specific bacteria. When reviewing the literature, we carefully consider the size of the groups examined and emphasize what biological material was used for the research. This is important in relation to the reliability of translating research from the laboratory to the clinic.

First, however, to introduce the topic, we will describe the lung microbiome, the history of research on it, and its normal composition.

## 2. Microbiome

The human body is an ecosystem for many microorganisms—the commensal, symbiotic, and pathogenic. Bacteria, archaea, fungi, protists, and viruses inhabiting particular regions, like the skin, oral cavity, nasal cavity, stomach, small intestine, large intestine, urinary tract, and vagina (hundreds of trillions of bacteria in number), constitute the microbiome of a given niche [1,2]. The term microbiome, although sometimes used interchangeably with microbiota, is a wider term, as it refers to the set of genomes of all the microorganisms within a specific environment. In turn, microbiota defines the set of microorganisms themselves.

Research on the human microbiome began in 2007 when the Human Microbiome Project (HMP) was launched. The main goal of the HMP has been to characterize the abundance, diversity, and functionality of the genes present in all microorganisms that permanently live in different human body sites: the human intestine, oral cavity, and skin [3,4]. Since the lungs were initially assumed to be sterile, the respiratory tract microbiome was investigated in-depth later in 2011 [5]. Then, work began to identify and analyze the lung microbiome and its role in health and disease and to develop new approaches for diagnosing and treating respiratory infections. The fact that microorganisms inhabit the human body has been known for a long time. However, the scale of this phenomenon, the number, and, above all, the diversity of these microorganisms was not realized. The results of the Human Microbiome Project consortium studies also demonstrated a high bacterial diversity and marked inter-individual variability at the species level in healthy individuals [4,6].

The success of understanding the human microbiome has been made possible by developing modern molecular techniques, which do not require cultivating bacteria using standard laboratory techniques [7,8,9]. The methods, including 16S rRNA gene sequencing and metagenomic analysis, have enabled the identification of these bacteria that cannot be detected using traditional, culture-based methods. Currently, the sequencing of the 16S rRNA gene, harbored by all bacteria, is widely used. Based on the homology of conservative regions in the 16S rRNA gene in different bacteria, it is possible to identify bacteria at the genus level. In contrast, metagenomic analysis allows for the analysis of all genetic information present in the microbiome population. The progress made in recent years in molecular technologies is remarkable. NGS (next-generation sequencing) of the 16S rRNA gene and metagenomic analysis has become more accessible, faster, and cheaper. This further improves microbiome research. These methods, referred to as culture-independent or molecular-based techniques, enable the identification and detection of bacteria by analyzing their DNA or other bacterial metabolites directly from clinical samples [9].

The interactions between the host and microbiota are established immediately after birth and constantly evolve throughout life due to the access and the influence of the microbial DNA, metabolites, RNA, and protein molecules. In general, the processes in which the microbiota is involved include the degradation of nutrients, the fight against invasion by xenobiotics, the elimination of pathogens, and the maturation of the host immune system [10,11].

It’s worth noting that the microbiota in different body parts, previously considered separate, are closely connected. Recent research has focused on the immunological interaction between the gut and lungs and found that the organism’s overall health influences the composition of its microbiota [12]. On the other hand, the diversity and stability of the microbiota are essential indicators of an individual’s overall health and physical condition [13,14,15].

## 3. Physiological Lung Microbiota

The fetal lung is sterile and appears to be colonized by microbes after birth. The first microorganisms that inhabit the neonatal mucosa are derived from the mother’s vagina (vaginally delivered babies) or skin (babies delivered via cesarean section) [16]. The neonatal microbial communities in different niches are homogeneous over several days to weeks before differentiating [17,18]. Interestingly, it takes only several hours to colonize the upper airways of the neonates, and the predominant phyla in the tracheal aspirates taken only several hours after birth are *Firmicutes* and *Proteobacteria*, in addition to *Actinobacteria* and *Bacteroidetes* [19]. Full-term infants experience a gradual development of their lower respiratory tract microbiota within a span of less than two months after birth [20]. The principal origin for the lung bacteriome in a healthy state is the microbiota genus originating from the oropharynx, representant of the *Prevotella* and *Streptococcus* species, an unclassified member of the family *Pasteurellaceae*, a *Fusobacterium* species, and a *Neisseria* species, present in the mouth, stomach, and lungs [21]. So, the lungs are no longer considered sterile, and numerous studies have shown that the lungs have a unique microbial community [22,23]. An island model of lung biogeography has been described—suggesting that the lungs can be viewed as an “ecological island” within the human body, separate from other microbial habitats like the oral cavity or gastrointestinal tract. The more distal lung bacterial communities are less diverse and less similar in composition to the upper respiratory tract communities [24].

However, the lung microbiota is still poorly understood. *Firmicutes* and *Proteobacteria* were the first bacteria phyla identified in the healthy lung using culture-independent techniques. The lower airway samples NGS analysis showed that bacteria from the phyla *Bacteroidetes* (in particular the genus *Prevotella*), *Firmicutes* (genera *Streptococcus*, *Veillonella*), *Proteobacteria* (genus *Acinetobacter*), and *Actinobacteria* (genus *Corynebacterium*) were most abundant in the lungs of healthy individuals [21,22,25,26,27]. Interestingly, it has been found that these genera might not be present in all healthy individuals. Human lung microbiomes can be classified according to the bacterial load and taxa. Two groups of so-called pneumotypes have been distinguished: the supraglottic predominant taxa (SPT) and background predominant taxa (BPT) pneumotypes. The SPT has a high bacterial load and is enriched with oral microorganisms such as *Prevotella* and *Veillonella*. In contrast, the BPT has a low bacterial load and background environmental taxa such as *Acidocella* and *Pseudomonas* [25].

Although at a low replication rate, a continual renewal of the lung microbiome in healthy individuals has been observed. Several factors that affect the lung microbiome composition have been distinguished and grouped: (1) microbial aspiration from the upper airways (both oropharynx and nasopharynx were described to be a source of aspirated microbes, but also from gastric content via gastro-oesophageal reflux and microaspiration); (2) microbial removal by mucociliary clearance and coughing; (3) host immune defense mechanisms (both adaptive and innate); and (4) pH, oxygen tension, temperature, and the concentration of nutrients, which may favor the growth of various microbes [25,26,28]. Some of those mechanisms can disturb lung microbiota homeostasis.

## 4. Lung Microbiome Dysbiosis

The microbiota has been associated with health, i.e., normal physiology and function. However, the increasing amount of evidence stresses that a loss of homeostasis, leading to a dysbiotic state, plays a crucial role in promoting the progression of a broad spectrum of diseases, including autoimmune diseases, obesity, mental disorders, and also cancer [12,13,14,29,30]. When defining dysbiosis, two basic concepts are discussed. The “imbalance” refers to a significant change in the microbiota composition. Still, sometimes, it pertains to specific adjustments to the ratios of organisms, leading to the loss of homeostasis. Less often, dysbiosis is regarded as “change,” which encompasses broad observations of alterations, along with the notion that these changes are linked to disease. The “change” has negative consequences for the host [31]. In dysbiotic lung microbiota, the balance between microbial immigration and elimination is disturbed. The loss of microbial diversity is observed, with a decreased abundance of symbiotic bacteria and a predominance of pathogenic bacteria. Such changes are associated with the progression of chronic lung diseases, such as chronic obstructive pulmonary disease (COPD), cystic fibrosis, asthma, and idiopathic pulmonary fibrosis [32,33]. Sometimes, specific changes refer to taxon shifts that may be as fine-grained as increases in different strains of *Escherichia coli* or as encompassing as changed proportions of its phylum of *Proteobacteria* [31].

The factors affecting respiratory dysbiosis are physiological and environmental, like diet, smoking, alcohol consumption, and air pollution [34,35,36]. They influence the respiratory epithelium, disturbing immune responses and changing the lung microenvironment, thus creating an ideal environment for dysbiotic microbial communities to thrive.

The harmful substances present in cigarette smoke and air pollution (mainly particulate matter—PM 2.5), apart from directly damaging cells and tissues in the respiratory system, lead to an increased production of reactive oxygen species (ROS) and cause an oxidant–antioxidant imbalance. Smoking, additionally, decreases the effectiveness of antioxidants like glutathione peroxidase (GPx), which help neutralize ROS and maintain the balance between oxidants and antioxidants [37,38]. An increasing number of free radicals can harm critical metabolic pathways within bacteria—also those naturally present in that niche—weakening their ability to survive and thrive in the lung environment. This disruption in microbial metabolism can alter the production of essential metabolites, enzymes, and signaling molecules, further contributing to dysbiosis [39]. Inflammation caused by oxidative stress can create a favorable environment for dysbiosis, as inflammatory mediators may impact the growth and survival of certain bacterial species, potentially favoring the proliferation of pathogenic bacteria and disrupting the normal microbial balance.

Additionally, the medications used for idiopathic and infectious lung diseases have been linked to increased respiratory tract dysbiosis. Oral antibiotics can effectively treat mild to moderate lung infections like community-acquired pneumonia, bronchitis, and COPD exacerbations. In chronic lung infections related to cystic fibrosis or bronchiectasis, inhaled antibiotics are often used. Antibiotics, delivered through inhalers or nebulizers, directly target the lungs, and the site of infection allows for a high drug concentration and reduces the risk of side effects from systemic exposure [40]. However, prolonged or repeated antibiotic use (independently of the administration route) alters the bacterial abundance, composition, and diversity, also creating an environment susceptible to opportunistic or antibiotic-resistant bacteria colonization. Particularly, antibiotics like penicillin, cephalosporins, or macrolides have been shown to imbalance the lung microbiota and increase the risk of lung cancer in humans [41,42]. However, it turned out that the effect of antibiotics on bacteria can be used cleverly. Le Noci et al. [43] revealed in animal studies that the manipulation of the microbiome—via the treatment using aerosolized antibiotics—led to a significant reduction in lung metastasis of B16 melanoma.

Antimicrobial peptides (AMPs) are a class of antibiotics produced by various living organisms, including bacteria, fungi, animals, and plants. Their positive charge and small size (<10 kDa) enable them to destabilize and penetrate the bacterial membrane (without the involvement of receptors) and then interact with the components inside the cell, ultimately leading to cell death [44]. The other activity of AMPs is related to their strong immunomodulatory functions that help inhibit excessive inflammation, promoting immune balance, which is beneficial for clearing bacterial infections [45]. On the other hand, combating bacterial lung infections with AMPs poses a risk of toxicity to the epithelium or inflammatory reactions in the lungs, worsening the initial condition, which can further disrupt the microbiome balance.

The physiological factors that underlie the dysbiosis of the lung microbiota are related to the entry, clearance, or local replication of microorganisms. Increased aspiration affects the amount, and upper airway dysbiosis affects the composition of the lung microbiome. For example, the genus *Prevotella*—anaerobic bacteria—is widely present in the oral cavity (dorsum and lateral sites of the tongue), saliva, and dental plaque of healthy subjects. While in the oral cavity, the presence of many *Prevotella* species accounts for the eubiosis state. In the case of its microaspiration to the lower respiratory tract, it can activate the pro-inflammatory T helper 17 cells (Th17) and subsequently the STAT3 pathway [46]. Some *Prevotella* species can also be transferred by swallowing to the GI tract and microaspiration to the trachea, bronchi, and lungs [47].

The decreased clearance in the respiratory tract can be caused by immune system dysfunction, changes in sputum composition, the remodeling of bronchial architecture, or physical barriers. The increased replication of bacteria in the area can be attributed to a higher intake of nutrients or an increased bacterial population, which could be caused by either a higher input or clearance deficiency [48].

Additionally, the prolonged use of mechanical ventilation may disrupt the microenvironment and result in dysbiosis. Particularly, invasive mechanical ventilation, commonly employed in intensive care units, can potentially disturb the natural lung microbiota. The endotracheal tube and ventilator circuit can facilitate the entry of bacteria into the lungs while simultaneously reducing the clearance abilities. Ventilator-associated pneumonia results in a change in the lung microbiome, with a shift towards a dominant bacterial pathogen (primarily *Proteobacteria*). It was demonstrated that the alpha diversity rapidly decreased after intubation and continued to drop with prolonged mechanical ventilation compared to the control patients who were healthy and unventilated [49,50].

## 5. Lung Oncobiome

The studies on colon cancer provided the most substantial evidence of a connection between dysbiosis and cancer. This was likely due to the microbiota’s impact on the host’s immune system, cellular pathways, and the induction of inflammation in the mucosa. Additionally, the metabolites and toxins produced by the microbiota can have an oncogenic activity, directly damaging the DNA [51,52]. There is still little research on the mechanisms linking the microbiota of the lower respiratory tract to lung cancer, regarding its initiation and progression.

One of the studies showing a potential connection between the airway microbiome and the development of lung cancer was the study Druzhinin et al. [53], which analyzed the frequencies of chromosomal aberration and micronuclei in relation to the bacterial composition. The study aimed to explore the relationship between the sputum microbiome and genetic abnormalities associated with LC, and revealed significant differences in the sputum beta diversity of the microbiome between LC patients and control subjects. A higher level of chromosomal aberrations in LC patients was associated with a higher sputum abundance of the *Bacteroides nordii; Lachnoanaerobaculum orale; Porphyromonas endodontalis; Mycoplasma zalophi; Prevotella intermedia; Prevotella histicola*, and *Campylobacter rectus* species. Some other studies performed in lung cancer patients compared to control subjects also indicated that the lung cancer-associated microbiota profile differed from that found in healthy controls. The gradual microbiota profile shift observed in a study by Liu XH et al. [54]—who analyzed airway brushing samples—from a healthy noncancerous site to a paired cancerous site suggested a change in the microenvironment along with the development of lung cancer. Yu et al. [55] performed an interesting study that analyzed non-malignant lung tissues (*n* = 165) obtained from patients with lung cancer. The core lung microbiota was composed of the following phyla: *Proteobacteria* (five genera: *Acinetobacter*, *Pseudomonas*, *Ralstonia*, and two unknown), *Firmicutes*, *Bacteroidetes*, and *Actinobacteria*. The results clearly indicated that lung tissue microbiota was distinct from the other body sites microbiota (oral cavity, nasal cavity, gut, skin, and vagina). Similar results were obtained by Liu Y et al. [56], who analyzed the non-malignant lung parenchyma microbiome in patients with lung cancer. The data of these studies suggested that the lung microbiota is unique; however, as explored in lung cancer patients, it may not be entirely applicable to healthy subjects.

Several studies showed a clear difference between paired normal and tumor lung tissues, indicating a reduction in bacterial richness and diversity in the lung tumor samples [55,57,58]. Additionally, a significantly lower microbial diversity in the non-small cell lung carcinoma (NSCLC) patients’ microbiome as compared to the controls was reported in different biological materials: in sputum [34,59] and in the airway brushing samples [54,60]. The opposite results were reported only in one study. Greathouse et al. [61] found an increased bacterial diversity in tumor and adjacent normal lung tissue compared to the controls.

Oncobiome is a name encompassing a community of microbes that have any influence on tumor development and progression. A growing body of research examines the link between lung bacteria species and lung cancer. The results of the study using **intraoperatively collected bronchial fluids** pointed to the role of microbes originating from the oral cavity in patients with lung cancer, namely, *Streptococcus*, *Veillonella*, *Gemella*, *Porphyromonas*, *Olsenella*, and *Eikenella* [62]. Another study involving LC patients revealed the significantly increased abundance in the **bronchoalveolar lavage fluid** (BALF) of two phyla, i.e., *Firmicutes* and *Saccharibacteria* (formerly known as TM7), and four genera, i.e., *Veillonella*, *Megasphaera*, *Atopobium*, and *Selenomonas* [63].. The results indicated differences in the bacterial communities of patients with lung cancer and those with benign mass-like lesions. The combination of the two genera, namely *Veillonella* and *Megasphaera*, was suggested as a potential biomarker for lung cancer. Another interesting finding was that smoking patients with lung cancer had a significantly higher ratio of *Firmicutes* to *Bacteroidetes* than non-smoking patients. A high prevalence of *Firmicutes* (*Streptococcus*) in heavy smokers with lung cancer was also found in another study [54]. Smoking, a recognized risk factor for lung cancer, disturbs the epithelial barrier integrity, thus increasing the risk of infections.

Similar results were obtained in the analyzed **airway brushing** samples from LC patients. Liu et al. [54] indicated an increasing trend for *Streptococcus* and *Neisseria*. At the same time, *Staphylococcus* and *Dialister* species gradually declined from non-cancerous to cancerous sites. Tsay et al. [60] reported that the lower airway brushes of lung cancer patients were enriched in *Streptococcus* and *Veillonella* compared to the benign lung disease patients and healthy controls.

In a pilot study by Cameron et al. [64] conducting a **sputum** analysis, the *Streptococcus viridans* was significantly higher in four patients diagnosed with lung cancer, while *Granulicatella adiacens* showed the highest level of abundance change. Moreover, the *G. adiacens* abundance in the LC samples was correlated with six other bacterial species: *Enterococcus* sp. 130, *Streptococcus intermedius*, *Escherichia coli*, *S. viridans*, *Acinetobacter junii*, and *Streptococcus* sp. 6. The authors concluded that the spontaneous sputum could be a viable source of bacterial biomarkers for LC status. Another small analysis performed in sputum found increased *Granulicatella*, *Abiotrophia*, and *Streptococcus* compared to the healthy controls [34]. The study was conducted on never-smoking subjects with and without lung cancer.

**The salivary microbiome** of NSCLC patients was also different from that of the controls, indicating the role of oral microbial dysbiosis. The study’s results by Yan et al. [65] revealed that *Capnocytophaga*, *Selenomonas*, and *Veillonella* were more abundant. In contrast, *Neisseria* was less abundant in both the squamous cell carcinoma (SCC) and adenocarcinoma (AD) patients than in the controls. The main finding in their work was that the significantly elevated levels of *Capnocytophaga* and *Veillonella* in the saliva samples in the NSCLC group might serve as potential biomarkers for NSCLC detection and classification [65]. The study performed by Yang et al. [66] involved non-smoking women with lung cancer and found that two genera, *Sphingomonas* and *Blastomonas*, were significantly increased in the patients, whereas *Acinetobacter* and *Streptococcus* were higher in the controls [66]. Another study on the salivary microbiome revealed increased levels of the phylum *Firmicutes* and its two genera, *Veillonella* and *Streptococcus*, and decreased relative abundances of *Fusobacterium*, *Prevotella*, *Bacteroides*, and *Faecalibacterium* [59]. The abundance of *Firmicutes* (with class *Bacilli*) was also observed in the saliva of NSCLC patients by Bingula et al. [57]. In the same study, the microbiome was also analyzed in BALF (obtained directly on an excised lobe), non-malignant, peritumoral, and tumor tissues from NSCLC patients. The obtained results revealed differences between the BALF and lung tissue microbiota, both in diversity and in taxonomy. The phylum *Proteobacteria* dominated in the tissue samples, while *Firmicutes* (with class Clostridia) was more abundant in the BALFs [57]. Gomes et al. [67] analyzed the microbiome profiles in BALF from LC patients and found that *Brevundimonas*, *Acinetobacter*, and *Propionibacterium* were more enriched in lung adenocarcinoma. In contrast, *Enterobacter* was more enriched in squamous cell carcinoma [67].

Recent research has identified notable differences in the microbiota depending on the histopathological types of lung cancer. Notably, in cases of adenocarcinoma, there is an increased prevalence of *Thermus* (*Thermi*) and a decreased prevalence of *Ralstonia* (*Proteobacteria*) when compared to squamous cell carcinoma [55]. However, non-malignant tissues—which comprised most of the analyzed samples—showed no significant differences regardless of the type of lung cancer diagnosed in the patient [55]. Another study by Greathouse et al. [61] found that *Acidovorax*, *Klebsiella*, *Rhodoferax*, *Comamonas*, and *Polarmonas* were more common in SCC tissue and absent in AD, which correlated with *Pseudomonas*. Smokers were found to have an increased abundance of *Acidovorax* and *Klebsiella*. In general, the research indicated that *Proteobacteria* tended to dominate in cancerous lung tissue samples [61]. This was also confirmed by Apopa et al. [68], who found differential abundances across four phyla: *Proteobacteria*, *Bacteroidetes*, *Actinobacteria*, and *Firmicutes* in NSCLC tissue. The analysis included three types of tissue samples: AD, SCC, and normal lung tissue, and revealed a higher relative abundance of *Cyanobacteria* in adenocarcinoma [68]. In a pilot study by Peters et al. [58], the overall microbiome composition did not differ significantly between the paired lung tumor (mainly lung adenocarcinomas) and normal tissue samples with an increased abundance of the family *Veillonellaceae* and decreased levels of the genus *Cloacibacterium* and family *Erysipelotrichaceae*. An interesting study was performed by Huang D. et al. [69], who compared the microbiome between SCC and AD in two different biological samples: bronchial washing fluid (BWF) and sputum. They found differences, revealing that the genera *Veillonella*, *Megasphaera*, *Actinomyces*, and *Arthrobacter* were significantly higher in AD than in SCC [69].

The disturbed airway and lung microbiota may have implications for patient prognosis. Peters et al. [58] found that a greater bacterial diversity and greater abundance of the families *Bacteroidaceae*, *Lachnospiraceae*, and *Ruminococcaceae* and genera *Bacteroides*, *Faecalibacterium*, *Roseburia*, and *Ruminococcus* in normal lung tissue were associated with reduced survival. Contrarily, a greater abundance of *Koribacteraceae* and *Sphingomonadaceae* was associated with increased survival [58]. In an earlier study, correlations between the genus *Thermus* and advanced-stage lung cancer (IIIB, IV) and a high *Legionella* amount in patients with metastases were observed, thus suggesting that these bacteria might play a role in tumor progression [55]. In another study, the term “recurrence-associated BAL microbiome signature” (RABMS) was proposed, encompassing 19 differentially abundant genera—identified in pre-surgery BALF from patients with stage I NSCLC [70]. The patients with RABMS + BAL fluid microbiomes had worse recurrence-free survival. When the microbiomes of LC patients with distant metastasis were compared, it was found that *Streptococcus* spp. was significantly lower in the patients with adenocarcinoma and distant metastasis. At the same time, the genera *Veillonella* and *Rothia* were considerably higher in the patients with squamous cell carcinoma and distant metastasis [69]. This points to the diverse roles that bacteria play in tumorigenesis for different subtypes of lung cancer, but also indicates that different genera are related to the lung metastasis state.

As mentioned earlier, the performed studies varied in the source where the lung microbiota was obtained for analysis, whether from saliva, sputum, BALF, and bronchial brushings or lung tissue, introducing potentially differing results. One of the most critical issues to be addressed in lung microbiome studies is the choice of clinical material for analysis. The authors of only one study compared their results and those of others. They concluded that bronchial washing samples might better reflect the lung microbiota of lung cancer tissues than sputum samples [69]. However, attention should be paid to the fact that the indirect methods for sampling the lungs, based on airway specimens such as BAL fluid, saliva, or sputum, may not accurately represent the lung microbiota due to contamination by the upper respiratory tract or oral microbiota.

Additionally, most of the studies were based on small sample sizes. The sample numbers varied from as low as four to eight subjects to not exceeding 50 patients in most studies. The compiled results of the studies conducted so far that have analyzed the microbiome in patients with lung cancer are presented in Table 1. It was indicated whether the study included a control group (healthy people or non-cancerous tissue samples) or a group with benign lesions. Printed in bold, *Veillonella* and *Megasphaera* in Lee’s study [63], *Prevotella*, *Veillonella* in Zeng’s study [71], and *Capnocytophaga* and *Veillonella* in Yan’s study [65] have been indicated by the researchers as potential biomarkers for NSCLC. However, more extensive studies are needed to validate these findings before they can be used as lung cancer biomarkers and included in therapeutic approaches.

The overwhelming number of studies indicated that *Streptococcus* and *Proteobacteria* may be the critical bacteria of lung cancer. However, additional extensive studies are necessary to confirm the microbial biomarkers specific to lung cancer patients and identify the most dependable biological sample for analysis, preferably non-invasive. Exhaled breath condensate (EBC), a biological fluid and natural matrix of the respiratory tract, appears to be a promising biological material for research in lung cancer patients. The EBC collection, which involves condensing the exhaled vapor, is a safe and non-invasive method that enables repeated sampling without causing any discomfort to patients [73]. The EBC analysis allows for the detection of bacterial or viral nucleic acids, as well as markers of inflammation and oxidative stress. However, it is difficult to exaggerate the superiority of this method over other, more invasive ones. Some studies showed a complete concordance between the bacterial pathogens cultured from BALF and EBC [74], but the results of others did not correlate well [75]. The reason for the differences may be related to the sampling place. In the case of the EBC, these are the central and peripheral airway compartments [76,77]. Glendinning et al. [77] compared the microbiota sampling methods, namely the invasive bronchoscopic procedure with a less invasive EBC collection, and demonstrated that the EBC samples contained significantly less bacterial DNA, suggesting that the EBC might be less sensitive for detecting lung microbiota, particularly those originating from biofilms adhered to the lung mucosa.

## 6. Mechanisms of Microbiome Influence on Lung Cancer Pathogenesis

In the process of carcinogenesis, a direct influence of bacteria has been suggested: (1) via regulating oncogenic signaling pathways in epithelial cells, thus leading to cell cycle disorder, mutagenesis, and DNA damage; (2) on the cells of the immune system, triggering an immune response, production and release of cytokines, thus changing the local immune microenvironment of the host; and (3) through MAMPs (microbe-associated molecular patterns), including the effects of bacteriotoxins, TLRs (toll-like receptors) signaling induction, and TNF (tumor necrosis factor) release. These mechanisms, of course, interact in the process of carcinogenesis. For example, in a mouse lung cancer model, administering an intranasal LPS (lipopolysaccharide, a membrane component of gram-negative bacteria) significantly enhanced pulmonary inflammation and lung tumorigenesis [78]. For another example, it has been found that several microorganisms (*Acidovorax*, *Klebsiella*, *Rhodoferax*, *Comamonas*, and *Polarmonas*) were more abundant in squamous cell carcinoma with *TP53* mutations in smokers [61]. It has been suggested that lung epithelial cells with *TP53* mutations due to tobacco smoke can be invaded by species that take advantage of the new microenvironment and may become tumor-foraging bacteria. Whether these bacteria induce mutations in *TP53* is currently under investigation. Interestingly, Jin et al. [79] found that germ-free or antibiotic-treated mice were significantly protected from lung cancer development due to *Kras* mutation and p53 loss.

### 6.1. Bacterial Toxins and Metabolites as Inflammatory Mediators of Lung Carcinogenesis

Certain bacterial toxins and metabolites lead to genetic mutations that promote uncontrolled cell growth and the formation of cancerous cells or create an environment conducive to tumor growth and a vicious cycle. Moreover, the molecules produced by bacteria might hinder the immune system’s capacity to detect and destroy cancer cells, which enables the cancer to evade immune surveillance and continue to grow [34]. One of the first bacterial infections linked to lung cancer was chronic chlamydiosis. Prolonged pneumocyte infection caused by *Chlamydia pneumoniae* has been linked to a 1.6 times higher risk of lung cancer development [60,80]. An in silico analysis demonstrated that *C. pneumoniae* proteins can target the mitochondria of host cells, causing its dysfunction. A study by Alshamsan et al. [81] found that 183 out of 1112 of these proteins target the mitochondria, while 513 target the cytoplasmic cellular processes. This, in turn, can disrupt normal cellular growth, alter apoptosis, and trigger lung carcinogenesis. These proteins can also be incorporated into intracellular organelles, forming a host cell proteome [82]. The *C. pneumoniae* oncogenic activity may be mediated by an endotoxin-like protein—the chlamydial heat shock protein-60 (cHSP60)—produced even during the chlamydia dormancy phase [83]. Heat shock proteins are a family of proteins found in all cells, highly conserved across species, including prokaryotes and eukaryotes, that play a crucial role in cellular stress responses. In the case of *Chlamydia*, cHSPs may resemble human HSPs, triggering autoimmune responses by activating the toll-like receptor 4 (TLR4) and inducing IL-6, TNF-α, and IL-1β production [82,83].

An example of a bacterial genotoxin is a cytolethal distending toxin (CDT) produced by various gram-negative bacteria, such as *Actinobacillus.* This bacterial toxin can directly cause DNA damage and trigger mutations [84,85]. It has been found in the human lung adenocarcinoma A549 cell line when a CDT induces apoptosis [86]. Another bacterial genotoxin is colibactin, produced by *Escherichia coli*, which can lead to double-strand breaks, resulting in a loss of genomic integrity and further cytotoxicity [84]. Among several *Pseudomonas aeruginosa*-identified toxins (Exotoxin A, U, Y, Exoenzyme S, T, Exolysin A, Alkaline Protease), the ExoS exhibits a genotoxin activity. An increased activity of the base excision repair enzyme OGG1 was observed in mice lung epithelial cells infected by *P. aeruginosa*. Additionally, it was proposed that ExoS, which has GTPase-activating and ADP ribosyl transferase activities, can directly cause DNA strand breaks via the phosphorylation of histone H2AX [84,87].

Cytotoxin-associated antigen A (CagA) is a virulence factor produced by *Helicobacter pylori* that, in the host epithelium, can modify various cellular signaling pathways and stimulate the toll-like receptors mediated response, i.e., the production of pro-inflammatory cytokines (IFN-α, IL-1, IL-2, IL-8) and chemokines leading to chronic inflammation. While *H. pylori* infection and CagA are primarily associated with stomach-related conditions (gastritis, peptic ulcers, and an increased risk of gastric cancer), studies demonstrated *H. pylori’s* presence in BALF and lung biopsies [88]. Another *Helicobacter* toxin, vacuolating cytotoxin A (VacA), named for its ability to form large, vacuole-like structures within the host cell, has immunomodulatory properties but also leads to mitochondrial membrane damage. The VacA exotoxin was detected in the bronchial epithelial cells of patients with interstitial pneumonia, where it activated the secretion of interleukins 6 and 8 [82,89]. *Streptococcus pneumoniae* is an opportunistic pathogen that produces a pneumolysin (PLY) toxin, which is a cholesterol-dependent cytolysin and a genotoxin. In the host tissue, PLY enables pore formation in the cell membrane, which can cause damage to the lung endothelium, as well as the nasal and tracheobronchial epithelium [90]. Targeting the host cell mitochondrial membrane, PLY can cause dysfunction and a loss of the mitochondrial membrane potential [91]. Moreover, the *S. pneumoniae* toxin can induce double-strand breaks in the DNA, thereby directly promoting genome instability and accelerating oncogenesis [92].

Through advanced sequencing techniques, scientists have discovered phyla and previously undetected species in human tissue, particularly in the lungs. A significant example was the detection of the *Cyanobacteria* phylum in lung cancer tissue, which is a gram-negative photosynthesizing bacterium [68]. It produces microcystin (MC)—a cyclic peptide toxin—that can induce mutations as predominantly large deletions, interfere with DNA damage repair processes, and finally contribute to genetic instability [93]. The increased expression of *poly (ADP-ribose) polymerase 1* (PARP1) was linked to a *Cyanobacteria* presence in the lung, and the *PARP1* expression was significantly increased in the adenocarcinoma lesion infected with *Cyanobacteria* compared to neighboring non-cancerous tissues [68]. PARP1, via the scavenger receptor CD36, can mediate inflammatory pathways that contribute to inflammation-associated lung carcinogenesis [68].

When pathogens and their metabolites interact with the host tissues, they can trigger inflammation by boosting the production of specific cytokines and inflammatory mediators, such as IL-1, IL-23, TNF, and IL-17. This happens through MAMPs, which then activate the critical downstream signaling pathways, like STAT3 and NF-kB pathways, as well as the ERK and PI3K pathways—regulating cell proliferation, survival, and differentiation—which are upregulated in lung cancer patients [94]. The evidence of the inflammation-associated carcinogenesis comes from the study of Tsay et al. [60], who found that the genera present in the respiratory tract, i.e., *Veillonella* and *Streptococcus* were associated with the upregulation of the ERK and PI3K signaling pathways in the airways of the patients with lung cancer. In the case of *Cyanobacteria* infection, the inflammation response included the secretion and activation of TNF, IL-1β, IL-4, and oncostatin M [68].

Triggering inflammation is even more visible in the case of well-known pathogenic factors, like *Mycobacterium tuberculosis.* In a systematic review by Liang et al., it was demonstrated that pre-existing tuberculosis is associated with a significantly increased lung cancer risk, particularly for the adenocarcinoma subtype [95]. Infections with *M. tuberculosis* activate the immune response mediated by the secretion of the interleukins IL-1,2,12, TNF-α, and INF-γ, and also trigger the neutrophils’ generation of reactive oxygen species. In turn, ROS can cause DNA breaks or interfere with mitochondria complexes, both contributing to cancer formation [82,96]. There was an association between *EGFR* mutation occurrence and lung AD in patients with pulmonary tuberculosis history [97].

Other bacteria that may affect lung cancer growth through the activation of inflammatory signaling is *Staphylococcus aureus*. *S. aureus* produces lipoteichoic acid (LTA) that induces a significant increase in cellular proliferation in NSCLC cell lines (A549 and H226), even at low concentrations [98]. LTA activates /increases the *IL-8* mRNA expression by binding to TLR-2, leading to cell proliferation. This finding suggests that *S. aureus* pulmonary infections may have a direct pro-proliferative effect on lung cancer growth. [94]. Another species of bacteria linked to lung cancer is gram-positive cocci *Granulicatella adiacens*, a bacteria previously considered part of the normal flora in the oral, intestinal, and respiratory tract. *G. adiacens* was significantly enriched in lung cancer patients’ sputum samples compared to healthy controls, suggesting its role in early NSCLC development [99]. The analysis of *G. adiacens* extracellular vesicles (EVs) proteome revealed the presence of many putative virulence factors, but not many associated with direct DNA damage. However, the presence of thioredoxin, which plays a crucial role in cellular redox regulation, may link *G. adiacens* to carcinogenesis in the lungs. Furthermore, the EVs of *G. adiacens* were found to be potent inducers of proinflammatory cytokines [100].

Reactive forms of oxygen (ROS) and nitrogen (RNS) are also compounds produced by the metabolism of bacteria. Free radicals contain unpaired electrons that enable the transfer of electrons to other molecules, harming amino acids, fatty acids, or even nucleic acids. If ROS and RNS accumulate excessively, they can cause biomolecules to undergo excessive oxidation, resulting in changes to protein structures and functions and further cellular damage and inflammation [37,101]. By directly damaging DNA or modifying cell signaling pathways, they create an environment favorable for carcinogenesis. Additionally, the inflammatory cells stimulated by dysbiosis can release reactive oxygen and nitrogen species, thus increasing their number and influence on angiogenesis and carcinogenesis [102,103]. Chronic *C. pneumoniae* infection may induce oncogenesis, also via the production and liberation of ROS, particularly nitric oxide during inflammation, thus leading to DNA damage [60]. The body’s inflammatory response to *C. pneumoniae* is associated with the production of cytokines such as IL-8, IL-10, and TNF in human alveolar macrophages and peripheral blood mononuclear cells [82,104]. It was demonstrated that *Cyanobacteria* toxin–microcystin also induces an excessive formation of reactive oxygen and nitrogen species, resulting in DNA damage [93]. The studies analyzing the bacterial toxin activity in lung oncogenesis are compiled in Table 2.

### 6.2. Modulation of the Adaptive Immunity by the Microbiota

Chronic inflammation is a known contributing factor to carcinogenesis. The underlying mechanisms include the induction of genomic instability, alterations in epigenetic events and subsequent aberrant gene expression, enhanced cell proliferation, and resistance to apoptosis (e.g., due to smoking). There is increasing evidence about the role of lung microbiota in lower airway inflammation. Additionally, molecules produced by bacteria might hinder the immune system’s capacity to detect and destroy cancer cells, which enables the cancer to evade immune surveillance and continue to grow [105]. As found in LC patients, lung microbiota from BALF enriched with supraglottic taxa was associated with a pro-inflammatory profile and the stimulation of Th17 cells, thus showing a pro-tumorigenic effect [25,46,60]. It was also confirmed by the more recent study by Tsay et al. [106], who showed that dysbiosis in the lower respiratory tract contributed to the inflammation of the tumor microenvironment characterized by an increased share of the Th1 and Th17 phenotypes.

IL-17-producing CD4 helper T cells (Th17 cells) are principal adaptive immune cells generated in the lung during inflammation. Th17 cells play a critical role in promoting chronic tissue inflammation through the upregulation of proinflammatory cytokines and chemokines in epithelial cells, where they are efficiently recruited upon inflammation [107]. As revealed by Chang et al. [108], in a mouse model of lung cancer, the role of Th17 cell-mediated inflammation was critical in tumorigenesis during the early stages of pulmonary adenocarcinoma. IL-17 accelerated cancer development, at least in part, by recruiting myeloid cells and promoting inflammation. The study of Wang et al. [109] revealed that Th17 cell-derived IL-17A played an essential role in the tumor progression of NSCLC via STAT3/NF-kB/Notch1 signaling. The authors clearly indicated that the levels of Th17 cells and IL-17A mRNA were increased in NSCLC patients (both in blood and cancerous lung tissue), and in vitro studies revealed that IL-17A could promote the invasion, migration, and cancer stem cell-like properties of NSCLC cells [109].

In NSCLC, the Janus kinase/signal transducers and activators of the transcription (JAK/STAT) pathway are crucial for promoting cellular survival and growth. The STAT protein family consists of seven transcription factors, including STAT3 and STAT5A/B, which have oncogenic properties both in vitro and in vivo [110]. While direct evidence linking dysbiosis and the STAT3 protein is limited, the activation through IL-17 may explain the interaction between dysbiosis, immune responses, and the activation of the activity of transcription factors like STAT3. Jin et al. [79], using germ-free preclinical lung cancer models, demonstrated that the lung commensal microbiome can induce the activation and proliferation of γδ T cells by stimulating myeloid cells to produce Myd88-dependent IL-23, IL-1β, and IL-17, and lead to a pro-inflammatory state that induces tumor proliferation. Another process linking dysbiosis to tumor cell proliferation is mediated by IL-22, whose production is activated in many infectious and inflammatory disorders. In mice lung cancer models, the presence of the Kras mutation led to the activation of the NF-κB pathway and to the secretion of IL-6 and IL-17, which was related to the increase in the IL-22 secretion [111]. CD4+ T helper lymphocytes (Th17, Th22, and Th1), natural killer T cells (NKT), innate lymphoid cells (ILC), and γδ T cells are all capable of producing cytokine IL-22 [112]. The dual action of IL-22 has already been proven in colorectal cancers, where IL-22 increases inflammation during the acute phase. Then, in the silencing phase, IL-22 promotes tissue repair, which can lead to the development of neoplastic lesions [113]. What is important is that cytokine IL-22 acts only on non-hematopoietic stromal cells, keratinocytes, hepatocytes, and particularly epithelial cells, i.e., in lungs and alveoli [112]. IL-22 operates through the heterodimer complex protein formed by the IL-22R1 and IL-10R2 coreceptors that phosphorylate the Stat3 protein. The phosphorylated Stat3 (P-Stat3) forms dimers and moves into the nucleus, leading to the activation of the STAT3 pathway downstream effectors [114]. The protein Stat3 plays a crucial role in the differentiation of Th17 and the production of T cell-dependent IgG responses. It also contributes to maintaining epithelial homeostasis, promoting wound healing, and producing antimicrobial factors such as β-defensins in response to infections caused by *Staphylococcus* and *Candida* [115]. Nevertheless, the IL-22 pro-inflammatory properties can also lead to harmful consequences, particularly when IL-22 is released alongside other pro-inflammatory cytokines, particularly IL-17 [112].

High IL-22 levels were detected in lung cancer patients, both in the primary lung tumors (bronchoalveolar lavage and bronchial washings) and in the circulating blood (in serum). The elevated immuno-expression of the IL-22 receptor R1 did not correlate with systemic inflammation but correlated with poor overall survival [116]. It was confirmed in adenocarcinoma patients with KRAS mutation, in whom the increased expression of the IL-22 receptor correlated with a worse prognosis, i.e., poor recurrence-free survival [111].

As found in animal studies, the genetic silencing of IL-22 (IL-22 knockout mice) has led to a strong decrease in tumor size [111]. Similarly, in the studied lung cancer model, the lack of IL-17 reduced tumor cell proliferation and angiogenesis and decreased the expression of proinflammatory mediators. It also reduced the recruitment of myeloid cells, which are recruited by IL-17, thus indicating that they play a pro-tumor role [108]. Based on their results, the authors suggested a novel way for the prevention and treatment of lung cancer via targeting Th17 cells or IL-17 in the early stage of lung cancer and COPD patients. The preclinical evidence obtained by other researchers [106]—in lower airway dysbiosis induced by aspiration of oral commensals—has confirmed that the direct blocking of the IL-17 pathway using anti-IL-17 monoclonal antibodies can slow tumor growth.

### 6.3. Modulation of the Innate Immunity by the Microbiota

The accumulating evidence indicates that bacterial MAMPs and TLRs contribute to carcinogenesis. Bacteria and their metabolites activate the TLR receptors on immune and epithelial cells, and thus induce inflammation. The cells of the innate immune response, as well as epithelial cells, express various receptors, such as TLRs, which inform about the invasion of pathogens. TLRs react to exogenous infectious ligands, such as cell wall elements, including LPS of gram-negative bacteria, lipoteichoic acids of gram-positive bacteria, motility systems, single- and double-stranded RNA, and DNA of microorganisms. The receptors of the TLR1 subfamily (TLR1, 2, 6, 10), TLR4, and TLR5 are found mainly in the cell membrane. They recognize the elements of cell walls, capsules, and movement apparatuses of pathogenic microorganisms. TLR3, TLR7-9, and TLR11-13 are found in intracellular organelles, such as endosomes, and are mainly involved in recognizing pathogen nucleic acids. Signal cascades are activated when the appropriate ligands are bound to the TLRs [117]. The activation of adaptive proteins begins, leading to the kinase cascades’ activation. The main signaling kinases are IRAK (interleukin-1 receptor-associated kinase), TRAF (TNF receptor-associated factor), MAPK (mitogen-activated protein kinase) and IKK (IκB kinase). The signal pathways triggered by TLRs lead to the activation of the transcription factors, such as NFκB (nuclear factor κ-light chain-enhancer of activated B cells) and AP-1 (activator protein-1) [117,118].

The above processes stimulate the expression and secretion of pro-inflammatory cytokines, such as TNF-α, IL-6, and IL-1beta chemokines. Several studies have indicated NFκB signaling as a mechanistic link between inflammation and lung cancer [119,120,121]. It has also been found that the expression of TLR4 was higher in lung cancer tissue than in paracancerous tissue [122], thus supporting the role of this receptor signaling in cancerogenesis [123,124]. Additionally, it has been clearly shown that IL-6 plays an essential role in lung cancer promotion [125], and its blockade significantly inhibits lung cancer development, STAT3 activation in tumor cells, tumor cell proliferation, and angiogenesis markers [126].

It is known that an upregulation of the ERK and PI3K pathways plays a central role in cell proliferation, survival, and tissue invasion at an early stage of lung cancerogenesis [94,127]. Their importance is enhanced by the possibility of being considered a therapeutic target [128,129]. The PI3K/AKT pathway is an intracellular signaling pathway that regulates various cellular functions, including cell survival, metabolism, and growth. Upon the binding of extracellular signaling molecules, the membrane receptor activation activates intracellular PI3K, which converts PIP2 lipids to PIP3. Then, AKT binds to PIP3, which can be phosphorylated by PDK1 and mTOR at two different sites, thus resulting in the full activation of AKT. pAKT phosphorylates several downstream molecules (BAD, IKK, CREB, FOXO1, and mTORC1), which regulate numerous cellular processes. The dysfunction of this pathway can lead to its over-activation and is associated with numerous pathological processes, including tumorigenesis [94]. As mentioned earlier, *Streptococcus* and *Veillonella*—found to be enriched in the lower airways of patients with lung cancer—are associated with an upregulation of the ERK and PI3K signaling pathways, and it was confirmed in vitro by the exposure of the airway epithelial cells to *Veillonella*, *Prevotella*, and *Streptococcus* [60,106]. The analysis showed that these oral taxa were associated with the upregulation of the PI3K/PTEN, ERK/MAPK, IL-6/IL-8, and p53 mutation pathways, thus indicating the mechanism linking the microorganisms and a cascade of events leading to lung cancer development.

## 7. Conclusions—Perspectives

Recent research has made significant progress in understanding the connection between the microbiota and lung cancer. It has been discovered that the microbiota of lung cancer patients differs from that of healthy individuals in terms of abundance and diversity. However, there is still much to learn about how changes in the composition and metabolism of the microbiome contribute to the development of lung cancer.

To gain a better understanding, it is crucial to conduct large-scale studies investigating the interaction between the microbiota and the carcinogenesis process in lung tissue. Key analysis-related issues must be addressed to accomplish these studies, including selecting clinical samples, DNA extraction methods, platforms for 16S rRNA gene sequencing, and improvements in analytical techniques. There is also a significant need for more research on the lung mycobiome and virome. These efforts will help to further understand the microbiota role in lung cancer and ultimately provide new insights regarding the microbiome-based prevention and treatment of lung cancer.

The field of lung microbiota is relatively new, and the number of relevant studies is still small, though comparing them is challenging due to varying methodologies and limited patient participation. Therefore, at the moment, it is difficult to identify those microorganisms that are unambiguously involved in oncogenic processes in the lungs. Distinguishing them from opportunistic organisms will pave the way for developing screening, diagnosing, and therapeutic modalities for lung cancer patients.

## Figures and Tables

**Table 1:** The summary of the relevant studies on the lung microbiome.

Sample Type	n (Lung Cancer)	Identified Bacteria	Control Included *	Benign Lung Lesions	Ref.
Airway brushings	39	*Veillonella* and *Streptococcus*	+	+	[60]
Airway brushings	24	↑ *Streptococcus*, *Neisseria*; ↓ *Staphylococcus*, *Dialister*	+	-	[54]
BALF	9	*Streptococcus*, *Veillonella*, *Gemella*, *Porphyromonas*, *Olsenella*, and *Eikenella*	-	-	[62]
BALF	20	Phyla: *Bacteroidetes*, *Proteobacteria*, *Firmicutes*, Genera: *Veillonella*, *Megasphaera*, *Atopobium*, *Selenomonas*	+	+	[63]
BALF	49	AD: ↑ *Brevundimonas*, *Acinetobacter* and *Propionibacterium* SCC: ↑ *Enterobacter*	+	-	[67]
BALF	91	Phyla: *Bacteroidetes*, *Proteobacteria*, *Actinobacteria*, *Firmicutes*; Genera: *Haemophilus*, *Prevotella*, *Propionibacterium*, *Pseudomonas*, *Rothia*, *Streptococcus*;	+	+	[72]
BALF	46	Phyla: *Firmicutes*, *Bacteroidetes*, *Fusobacteria*; Genera: *Actinomyces*, *Alloprevotella*, *Neisseria*, *Porphyromonas*, *Prevotella*, *Streptococcus*, *Veillonella*;	-	+	[71]
Lung tissue	31	AD vs. SCC: ↑ *Thermus*, ↓*Ralstonia*;	+	-	[55]
Lung tissue	143	SCC: *Acidovorax*, *Klebsiella*, *Rhodoferax*, *Comamonas*, *Polarmonas*; AD: *Pseudomonas*;	+	-	[61]
Lung tissue	21	*Bacteroidetes*, *Proteobacteria (Actinobacteria*, *Firmicutes*, *Cyanobacteria*, *Acidobacteria*, *Chloroflexi)*; AD vs. SCC: ↑*Cyanobacteria*;	+	-	[68]
Lung tissue	30	↑*Firmicutes: Streptococcus*; *Bacteroidetes: Prevotella*; ↓ *Proteobacteria: Acinetobacter*, *Acidovorax*;	+	-	[56]
Lung tissue	19	↑*Veillonellaceae*; ↓*Cloacibacterium*, *Erysipelotrichaceae*;	+	-	[58]
Saliva	20	↑ *Veillonella*, *Capnocytophaga*, *Selenomonas*; ↓ *Neisseria*;	+	-	[65]
Saliva	39	↑ *Firmicutes: Veillonella* and *Streptococcus*; ↓ *Fusobacterium*, *Prevotella*, *Bacteroides*, *Faecalibacterium*;	+	-	[59]
Saliva	75	↑ *Sphingomonas* and *Blastomonas*;	+	-	[66]
Sputum	8	*Granulicatella*, *Abiotrophia* and *Streptococcus*;	+	-	[34]
Sputum	4	↑ *Granulicatella adicens*, *Streptococcus intermedius*, and *Mycobacterium tuberculosis*;	+	-	[64]
Bronchial washing fluid Sputum	40 52	Phyla: *Firmicutes* and *Proteobacteria*; Genera: *Prevotella*; Phylum: *Firmicutes*; Genera: *Streptococcus*;	-	-	[69]
BALF Saliva Tissue	18 18 18	*Firmicutes (Clostridia)*; *Firmicutes (Bacilli)*; *Proteobacteria*;	-	-	[57]

* non-malignant group included ↑ = increased; ↓ = decreased—as compared to controls; AD—lung adenocarcinoma; BALF—bronchoalveolar lavage fluid; SCC—lung squamous cell carcinoma.

**Table 2:** The summary of toxins directly related to the lung carcinogenesis process.

Identified Bacteria	Toxin/ROS	Biological Effect	Ref.
*Actinobacillus*	CDT	Direct DNA damage and triggering mutations	[84,85]
*Chlamydia pneumoniae*	ROS	Oxidative stress, DNA damage, mutagenesis	[60,80]
	?	Targeting mitochondria, cellular redox regulation	[81,82]
	cHSP60	Triggering autoimmune responses by activating the toll-like receptor 4	[82,83,104]
*Cyanobacteria*	MC	Generation of reactive oxygen species, DNA damage, mutagenesis	[93]
		Contributing to the inflammation-associated lung carcinogenesis—activation of TNF, IL-1β, IL-4, and oncostatin M	[68]
*Escherichia coli*	Colibactin	Direct DNA damage (double-strand breaks) promoting genome instability	[84]
*Granulicatella adiacens*	?	Activation of proinflammatory cytokines secretion	[99,100]
	Thioredoxin/ROS	Targeting mitochondria, oxidative stress, and cellular redox regulation	[100]
*Helicobacter pylori*	CagA	Triggering autoimmune responses by activating the toll-like receptor, increased secretion of pro-inflammatory cytokines and chemokines	[88]
	VacA	Increased secretion of IL-6 and Il-8	[89]
		Targeting mitochondria, cellular redox regulation	[82]
*Mycobacterium tuberculosis*	ROS	Oxidative stress, DNA damage, mutagenesis	[96,97]
	?	Increased secretion of cytokines, IL1,2.3,4,10,12,14, 17, IFN-γ and TNF-α	[82]
*Pseudomonas aeruginosa*	ExoS	Direct DNA damage (double-strand breaks) promoting genome instability	[84,87]
*Staphylococcus aureus*	LTA	Triggering autoimmune responses by activating the toll-like receptor 2, leading to cell proliferation	[98]
*Streptococcus pneumoniae*	Ply	Activation of the ERK and PI3K signaling pathways	[60,90]
		Direct DNA damage (double-strand breaks) promoting genome instability	[91]
		Targeting mitochondria, cellular redox regulation	[92]
*Veillonella*	?	Activation of the ERK and PI3K signaling pathways	[60]

CagA—Cytotoxin-associated antigen A; CDT—Cytolethal distending toxin; cHSP60—Chlamydial heat shock protein-60; ExoS—Exotoxin S; LTA—Lipoteichoic acid; MC—Microcystin; Ply—Pneumolysin; ROS—Reactive oxygen species; VacA—Vacuolating cytotoxin A.

## Abbreviations

AD—Lung Adenocarcinoma; AMPs—Antimicrobial peptides; BALF—Bronchoalveolar lavage fluid; BPR—Background predominant taxa; BWF—Bronchial washing fluid; CagA—Cytotoxin-associated antigen A; CDT—Cytolethal distending toxin; cHSP60—Chlamydial heat shock protein-60; COPD—Chronic obstructive pulmonary disease; EBC—Exhaled breath condensate; ExoS—Exotoxin S; EVs—Extracellular vesicles; GI tract—Gastrointestinal tract; GPx—Glutathione peroxidase; HMP—Human Microbiome Project; HSP—Heat shock protein; IKK—IκB Kinase; IRAK—Interleukin-1 receptor-associated kinase; JAK/STAT—Janus kinase/signal transducers and activators of the transcription; LC—Lung cancer; LPS—Lipopolysaccharide; LTA—Lipoteichoic acid; MAMPs—Microbe-associated molecular patterns; MAPK—Mitogen-activated protein kinase; MC—Microcystin; NFκB—Nuclear factor κ-light chain-enhancer of activated B cells; NGS—Next-generation sequencing; NSCLC—Non-small cell lung carcinoma; PARP1—Poly(ADP-Ribose) polymerase 1; PLY – Pneumolysin; PM 2,5—Particulate matter; RABMS—Recurrence-associated BAL microbiome signature; ROS—Reactive oxygen species; RNS—Reactive forms of nitrogen; SCC—Lung squamous cell carcinoma; SPT—Supraglottic predominant taxa; TLR—Toll-like receptors; TNF—Tumor necrosis factor; TRAF—TNF receptor-associated factor); VacA—Vacuolating cytotoxin A.
